# Automated video-based AVPU assessment within a FHIR-enabled clinical decision support framework

**DOI:** 10.1038/s41598-026-57122-z

**Published:** 2026-06-22

**Authors:** João C. Ferreira, Pedro Faria

**Affiliations:** 1 ISCTE – Instituto Universitário de Lisboa, Lisbon, Portugal; 2 Molde University College, Molde, Norway; 3 Inesc Inov, Lisbon, Portugal

**Keywords:** AVPU scale, Consciousness assessment, Clinical decision support system, Computer vision, HL7 FHIR, Emergency care, Edge computing, Technology adoption modelling, Business and industry, Computational biology and bioinformatics, Health care, Mathematics and computing, Medical research

## Abstract

Accurate and timely assessment of consciousness is critical for triage, escalation of care, and patient safety in emergency and hospital settings. However, documentation using the AVPU scale (Alert, Verbal, Pain, Unresponsive) remains inconsistent owing to high workload, subjectivity, and fragmented workflows. This study developed and evaluated Consc.ia, a video-based clinical decision-support platform that automates AVPU inference while preserving clinician oversight and enabling seamless, interoperable documentation through HL7 FHIR. A simulated AVPU dataset comprising 136 videos from 58 healthcare professionals (physicians, nurses, paramedics, and first responders) was created under controlled conditions with ethics approval from the ISCTE – Instituto Universitário de Lisboa Ethics Commission (reference CE-ISTA/2025.08, July 2025). The system architecture combines edge-computing computer vision for real-time extraction of facial landmarks, eye state, arm movement, and verbal responses; a clinician-in-the-loop validation layer; and FHIR-mapped Observation resources for direct EHR integration. Three deployment scenarios (Emergency Medical Services, Emergency Departments, and Intermediate Care wards) were designed and compared. Technology adoption was modelled using Rogers’ Innovation Adoption Curve and the Bass Diffusion Model (*p* = 0.01, q = 0.35, M = 111 Portuguese hospitals). The architecture achieves low-latency inference with privacy-by-design (local processing, no raw video storage). Stakeholder validation confirmed strong workflow fit and highlighted persistent documentation gaps during EMS-to-hospital transitions. Scenario analysis revealed distinct hardware and integration requirements (ambulance edge device versus ward multi-camera server). Bass modelling projects gradual adoption, reaching approximately 50% of Intermediate Care wards by 2037 in the realistic scenario, with the “chasm” phase occurring between 2030 and 2032. Sensitivity analysis identified early clinical evidence and FHIR integration support as the strongest accelerators of diffusion. As this constitutes a proof-of-concept study, no quantitative AVPU classification metrics (e.g., accuracy, sensitivity, specificity, or confusion matrix) are reported at this stage; empirical model evaluation against expert-annotated clinical recordings is identified as the primary prerequisite for future validation and clinical translation. As a proof-of-concept that has not yet undergone clinical validation, Consc.ia offers a feasible, interoperable solution for standardising AVPU documentation and strengthening early warning systems. By combining video analytics, edge computing, clinician validation, and FHIR integration, the platform addresses a longstanding gap in emergency-care digitalisation and provides a clear roadmap for real-world adoption.

**Supplementary Information:**

The online version contains supplementary material available at https://doi.org/10.1038/s41598-026-57122-z.

## Introduction

Emergency medical care—whether delivered in the pre-hospital environment by Emergency Medical Services (EMS) or within hospital healthcare facilities—depends on rapid, reliable, and standardised assessment of patient severity. Early identification of clinical deterioration is essential for enabling timely interventions, guiding resource allocation, and improving patient outcomes in healthcare settings^1^. To support this process, triage protocols such as the Manchester Triage System (MTS)^2,3^ and the National Early Warning Score (NEWS/NEWS2)^4^ have become foundational tools across healthcare systems worldwide. These protocols combine physiological measurements with structured clinical observations to generate a priority score, ensuring that the most critical patients receive immediate attention.

A key component of these triage mechanisms is the evaluation of a patient’s level of consciousness (CL) or altered mental status (AMS). Consciousness assessment is one of the strongest predictors of acute deterioration, hospital mortality, and the need for rapid response interventions^5^. Despite its prevalence^6^ and importance, consciousness evaluation is often performed inconsistently, documented incompletely, or omitted entirely from electronic records^7,8^. This issue is especially problematic in systems such as NEWS, where the AVPU scale (Alert, Verbal, Pain, Unresponsive) (ATLS Subcommittee)^9^, is the only score that requires subjective human interpretation, while all other vital signs are obtained through automated medical devices.

Simultaneously, healthcare systems worldwide are undergoing a gradual transition toward continuous, data-driven monitoring supported by digital technologies. Wearable sensors, portable medical devices, and integrated physiological monitors increasingly provide real-time streams of vital signs and patient outcomes^10–13^. However, consciousness assessment has not yet benefited from equivalent technological innovation, remaining entirely dependent on human judgment. This contrast highlights a significant gap in automation, reliability, and interoperability within modern emergency care workflows.

Although consciousness assessment is essential for clinical decision-making, its documentation remains incomplete or absent in a substantial proportion of triage interactions^14–16^. The reasons are multifactorial: time pressure, high workload, variability in staff experience, ambiguous qualitative descriptors, and the manual nature of the AVPU process^17–19^. Unlike heart rate, blood pressure, or oxygen saturation—which are captured by medical devices—AVPU requires clinicians to stimulate and interpret patient responses in real time.

The absence of an AVPU value compromises the reliability of NEWS-based risk stratification and may delay recognition of patient deterioration. In addition, incomplete consciousness assessments hinder communication between EMS teams and hospital staff during patient handover, contributing to information gaps at a critical transition point in emergency care, and during processual assessments at the ED and Intermediate Wards.

At the same time, no widely adopted technological solution currently exists for automated or semi-automated consciousness assessment and research in this domain remains sparse.

A review of the scientific literature reveals a lack of comprehensive solutions addressing the automation of consciousness assessment:

- Scarcity of applied research: Only one known study explores video-based estimation of consciousness, and it remains limited in scope and external validity^20^;
- Absence of integration into clinical workflows: No existing work describes how automated AVPU classification could be embedded into EMS systems, ED triage software, or inpatient electronic health record (EHR) systems;
- No interoperable data architecture: There is no detailed proposal linking video inference outputs to standardised healthcare data formats such as FHIR;
- No analysis of adoption feasibility: The diffusion of such technology in hospitals and EMS services has never been modelled or contextualised using established innovation adoption frameworks;
- Lack of real-world data: Real clinical video datasets of AVPU procedures do not exist due to ethical and logistical constraints, requiring new controlled data collection efforts.

This work investigates how video-based analytics can support more consistent and interoperable documentation of patient consciousness in emergency and hospital care, while advancing from a proof-of-concept prototype toward a product-oriented solution. The research questions are structured to guide the analysis from clinical requirements to technical feasibility, architecture and interoperability design, scenario-based deployment, and adoption considerations for the proposed prototype, Consc.ia.

**RQ1 — Clinical and operational requirements.** What clinical and operational requirements must be satisfied to improve the consistency and timeliness of AVPU consciousness assessment documentation across EMS-to-hospital care transitions?

**RQ2 — Technical feasibility of video-based inference.** To what extent can video-based analytics provide feasible inference of AVPU-related responsiveness under real-time and environmental constraints typical of emergency and hospital settings?

**RQ3 — Product-oriented system architecture.** What end-to-end architecture is required for Consc.ia to operate as a safe and usable clinical decision support prototype, including clinician validation, auditability, and privacy-by-design?

**RQ4 — Interoperability and structured documentation.** How can AVPU outputs generated by Consc.ia be represented and exchanged using HL7 FHIR to support interoperable integration with triage processes, NEWS/NEWS2 calculation, and EHR documentation workflows?

**RQ5 — Deployment scenarios and operational fit.** How do deployment requirements and constraints differ across EMS, Emergency Departments (ED), and Intermediate Care (IC) wards, and which implementation configurations best support the progression of Consc.ia from prototype toward operational readiness?

**RQ6 — Adoption and productisation pathway.** What adoption trajectory can be anticipated for a video-based AVPU decision support product such as Consc.ia within the healthcare ecosystem, and which factors most strongly influence diffusion and scale-up according to innovation diffusion models?

These research questions ensure a coherent alignment between the clinical problem, the proof-of-concept implementation, and the product development pathway explored throughout the work. It is important to note explicitly that the present work is a proof-of-concept and architecture feasibility study, not a clinically validated system. No quantitative model performance metrics are reported, because the system has not yet been evaluated on real patient data. The contribution is the demonstration of technical and workflow feasibility, the design of an interoperable architecture, and the identification of the research and validation pathway required to progress toward clinical deployment.

This research work is developed at the intersection of digital health, applied artificial intelligence, and emergency care workflow modernisation. The work is motivated by real-world constraints observed in time-critical clinical environments, where rapid assessment protocols must be executed under high cognitive load, limited resources, and fragmented information flows between pre-hospital and in-hospital settings.

Within this context, the proposed Consc.ia prototype is positioned as a product-oriented clinical decision support capability designed to strengthen the quality and consistency of consciousness assessment documentation using the AVPU scale. The research context is defined by four complementary dimensions:

1. Emergency care operational context: This work targets environments such as EMS, Emergency Departments, and Intermediate Care wards, where consciousness assessment is clinically relevant, but documentation may be incomplete, delayed, or inconsistently recorded due to operational pressure and workflow variability.
2. Technology and feasibility context: The work assumes realistic constraints regarding camera positioning, lighting variability, patient movement, staff interference, and computational limitations, thereby motivating a design that supports real-time inference and practical deployment pathways.
3. Interoperability and health information systems context: The solution is designed with structured and interoperable documentation in mind, enabling the representation of consciousness assessments through HL7 FHIR and supporting integration into EHR workflows and early warning systems such as NEWS/NEWS2.
4. Product development and adoption context: The research is conducted with a clear transition path from proof-of-concept to product, considering governance, privacy-by-design, clinician validation, and adoption dynamics necessary for scaling into operational healthcare settings.

Medical devices and clinical support tools are subjected to rigorous regulatory scrutiny and risk-based classification under the Medical Device Regulation (MDR) framework within the European Union (Guidance on Qualification and Classification of Software in Regulation (EU) 2017/745 – MDR and Regulation (EU) 2017/746 – IVDR, n.d.). This is a mandatory process for commercialisation produced during more mature stages of industrial development. Involves a formal relation with a MDR Accredited Body to pursuit conformity assessment and certification under ISO13485. This thesis will not approach this aspect, although many conformity requirements are provided in safety-by-design and privacy and ethical concerns.

This research context frames this work as an applied and translational effort, bridging clinical workflow challenges and technological feasibility, while supporting longer-term innovation and commercialisation potential in emergency care decision support systems.

## Literature review

Below is a PRISMA flow diagram in tabular representation, see Table 1^21^.

- **Date range**: January 2000 – December 2024.
- **Outcome**: Only one study met all inclusion criteria, highlighting limited evidence in automated video-based consciousness assessment.
- No use of interoperability standards such as FHIR.

**Table 1:** PRISMA flow diagram.

Phase	Description	Records (*n*)
Identification	Records identified through database searching (PubMed, Scopus, Web of Science, IEEE Xplore)	214
	Duplicate records removed	63
Screening	Records screened (title/abstract)	151
	Records excluded	138
	– Not video-based methods	97
	– No consciousness assessment outcome	41
Eligibility	Full-text articles assessed for eligibility	13
	Full-text articles excluded	12
	– Video used only for documentation	7
	– No clinical validation	4
	– Non-human study	1
Included	Studies included in qualitative synthesis	1
	Included study	Choi^20^,

Only one peer-reviewed study to date has attempted to classify consciousness levels using computer vision^20^. While necessary as a proof of concept, its limitations include:

Absence of real clinical deployment;

Constrained dataset size;

No integration with triage protocols;

No linkage to NEWS scoring;

No consideration of EMS or ward environments;

This gap underscores the novelty of developing a system that can be deployed in operational environments and integrated with existing digital health infrastructures.

Clinical adoption requires more than an accurate model. Healthcare environments introduce specific challenges:

- Variable lighting and camera placement;
- Patient positions that differ from training data;
- Movement, agitation, or obstructions;
- Strict privacy regulations;
- Need for clinician confirmation of algorithm output;
- Requirement for seamless integration with EHR systems.

The lack of a complete architectural proposal addressing these constraints underscores the need for the work developed in this thesis^22,23^.

Computer vision systems can process video streams using either local (edge) devices or remote servers. In healthcare, especially EMS and emergency contexts, local processing presents several advantages^24^:

- Minimal latency during triage;
- Resilience to connectivity disruptions;
- Enhanced privacy, as raw video does not need to leave the local device;
- Lower dependence on hospital network stability;
- Immediate availability of results for documentation and decision-making.

However, centralised processing can support heavier models and benefit from shared computational resources. Choosing between local and centralised architectures depend on the clinical scenario, technical resources, and information governance constraints.

Edge inference devices suitable for AVPU classification include:

- Embedded GPU platforms (e.g., NVIDIA Jetson Orin Nano, delivering up to 40 TOPS at under 10 W, well-suited for ambulance deployment);
- Compact CPUs optimised for neural inference (e.g., Raspberry Pi 5 paired with an AI HAT+ accelerator, adequate for ward-based intermittent assessment);
- Integrated smart cameras with on-board processing (e.g., Luxonis OAK-D Pro, supporting on-device execution of lightweight YOLO or MobileNet-SSD variants at 30 fps).

These systems support optimised models and can operate within ambulances, triage rooms, or ward ceilings. Their ability to run inference without cloud connectivity is essential for EMS environments, where cellular networks may not guarantee consistent bandwidth.

Interoperability and Health Information Systems, the Electronic Health Records and Triage Systems. Hospitals and EMS organisations rely on complex information systems^25,26^, including:

- Electronic Health Records (EHR);
- Emergency Department Information Systems (EDIS);
- EMS coordination and dispatch platforms;
- Ward-level monitoring dashboards;
- Intensive care unit monitoring suites;
- Radiology and imaging systems such as PACS.

A video-based AVPU classifier must operate within this ecosystem. Automation alone is insufficient - the system must interact with broader clinical workflows.

Interoperability is central to integrating new technologies into healthcare systems. The Fast Healthcare Interoperability Resources (FHIR) standard provides structured formats for exchanging clinical data^27^. A video-based AVPU output can be represented using:

- Observation resources for consciousness assessment;
- Device resources describing cameras or processing units;
- Encounter resources linking to EMS or hospital episodes.

Embedding the classifier within FHIR-based exchanges ensures compatibility with national and international health information infrastructures.

Data Security, Ethics, and Privacy Considerations. Healthcare video data introduces unique privacy and security concerns. Any deployment must include^28,29^:

- Encrypted data transmission;
- Strict access controls;
- Minimisation of raw video storage;
- Clear consent mechanisms when applicable;
- GDPR-compliant processing;
- Robust audit logging;
- Options for de-identification where feasible.

Edge-processing architectures help mitigate privacy concerns by ensuring raw video remains locally processed and only numerical results (e.g., AVPU classification, timestamps, other metadata) are transmitted.

Ethical challenges include transparency of model decisions, clinician oversight, bias management, and the need to preserve clinician autonomy. Automated AVPU classification is intended as a decision-support tool and must always allow human validation or override.

Computer vision has matured into a reliable and widely adopted technology across healthcare settings, particularly in diagnostic imaging and behavioural analysis. Its capabilities in facial landmark detection, pose estimation, and real-time video processing provide a strong foundation for automated consciousness assessment.

Despite these advancements, research on automated AVPU classification remains extremely limited, with only one preliminary study addressing the problem. Real-world deployment introduces specific constraints that require careful architectural design, including edge computing, interoperability with EHR systems, and strict privacy protections. Adjacent research domains provide important context. Several groups have explored automated scoring of the Glasgow Coma Scale (GCS) using computer vision and physiological signals, demonstrating that individual GCS sub-components can be estimated from video with moderate accuracy under controlled conditions; their experience with training-data scarcity and domain shift is directly applicable to AVPU automation. Multimodal clinical AI systems fusing video with audio, wearable sensors, and EHR streams have shown improved robustness in detecting patient deterioration. Behavioural analysis using computer vision -- including pain detection from facial action units and agitation monitoring -- provides validated feature-extraction pipelines adaptable for AVPU-relevant cues such as eye opening and purposeful movement^30^. Situating Consc.ia within these adjacent domains strengthens its conceptual grounding and highlights validated methodological pathways for future empirical development.

These technological considerations provide the basis for the next chapter, which presents a comprehensive architecture for integrating automated AVPU classification into EMS and hospital workflows.

## Consc.ia: requirements and architecture

### Requirements elicitation and stakeholder-derived outcomes

The design of Consc.ia followed a product-oriented development approach in which system requirements were continuously refined through feedback from relevant stakeholders. Requirements elicitation combined (i) informal and semi-structured interviews with healthcare professionals and (ii) broader validation through an academic innovation environment involving a master-level population. This approach was adopted to ensure that the proposed architecture aligns with real-world constraints related to usability, workflow integration, interoperability, and privacy.

Throughout the development process, multiple informal and semi-structured interactions were conducted with healthcare professionals representing different operational contexts and roles, including physicians, nurses, paramedics, first responders, and healthcare consultants. These interactions were used to validate the problem relevance—namely the inconsistent documentation of consciousness assessment—and to identify architecture-level requirements that could enable real-world feasibility.

The most recurring requirements extracted from stakeholder feedback can be summarised as follows:

- R1 — Workflow neutrality: The system must not add cognitive burden during time-critical assessment and must operate as a supportive layer rather than a replacement for clinical judgement.
- R2 — Clinician-in-the-loop validation: AVPU outputs should be treated as decision support recommendations, requiring confirmation by a clinician prior to becoming part of the clinical record.
- R3 — Low-interaction user interface: The interface must be designed for minimal interaction (one-step confirmation, default suggestions, rapid override).
- R4 — Real-time feasibility: Processing latency must be compatible with emergency workflows, supporting rapid assessment and documentation near the point of care.
- R5 — Privacy-by-design: Video data should be minimised, with preference for transient processing and reduced retention of raw video content whenever possible.
- R6 — Auditability and traceability: Every AVPU output should preserve timestamp and contextual metadata, enabling accountability and clinical traceability.
- R7 — Interoperability-first integration: Outputs must be representable using structured standards (HL7 FHIR), enabling integration into EHR documentation and early warning scoring processes such as NEWS/NEWS2.

These requirements informed both the architectural choices (e.g., edge processing feasibility and modular pipeline) and the functional constraints of the decision support layer.

To broaden validation beyond direct stakeholder interviews and to collect additional requirements at scale, Consc.ia was also discussed in an academic innovation context involving a master-level cohort. During the ManagiDiTH Summer Week, the author presented the clinical relevance of consciousness assessment and the AVPU documentation gap, proposing it as a healthcare business challenge that could be approached through applied digital innovation.

The pitch and subsequent discussions were delivered to an audience of approximately 180 master-level students, attending as part of a structured educational and innovation environment. The cohort included participants with exposure to healthcare services, information systems, and digital health challenges. Feedback was collected during the session and reinforced through later interactions with participants and attendees involved in healthcare operations, including clinical and IT roles.

Although this cohort does not represent end-users in the strict clinical sense, the master population acted as a structured validation mechanism for:

- Testing whether the problem statement is understandable and operationally credible,
- Identifying expected deployment constraints (workflow, connectivity, governance),
- Clarifying requirements for integration, data handling, and interface simplicity,
- Supporting prioritisation of features for early product maturity.

This activity also supported the refinement of Consc.ia as a product-oriented solution by reinforcing that adoption is dependent not only on model performance, but also on its alignment with clinical practice, interoperability requirements, and governance constraints.

Based on the combined feedback sources, the final architecture presented in this chapter was defined to operationalize the following design principles:

- Decision support rather than automation, ensuring clinical control and safety.
- Minimal interaction confirmation, to match emergency care time constraints.
- Interoperable documentation, enabling structured AVPU capture using HL7 FHIR.
- Privacy-by-design processing, limiting exposure and retention of raw video.
- Deployment realism, supporting EMS and hospital contexts without requiring major infrastructure changes.

### Architecture

**Fig. 1:**
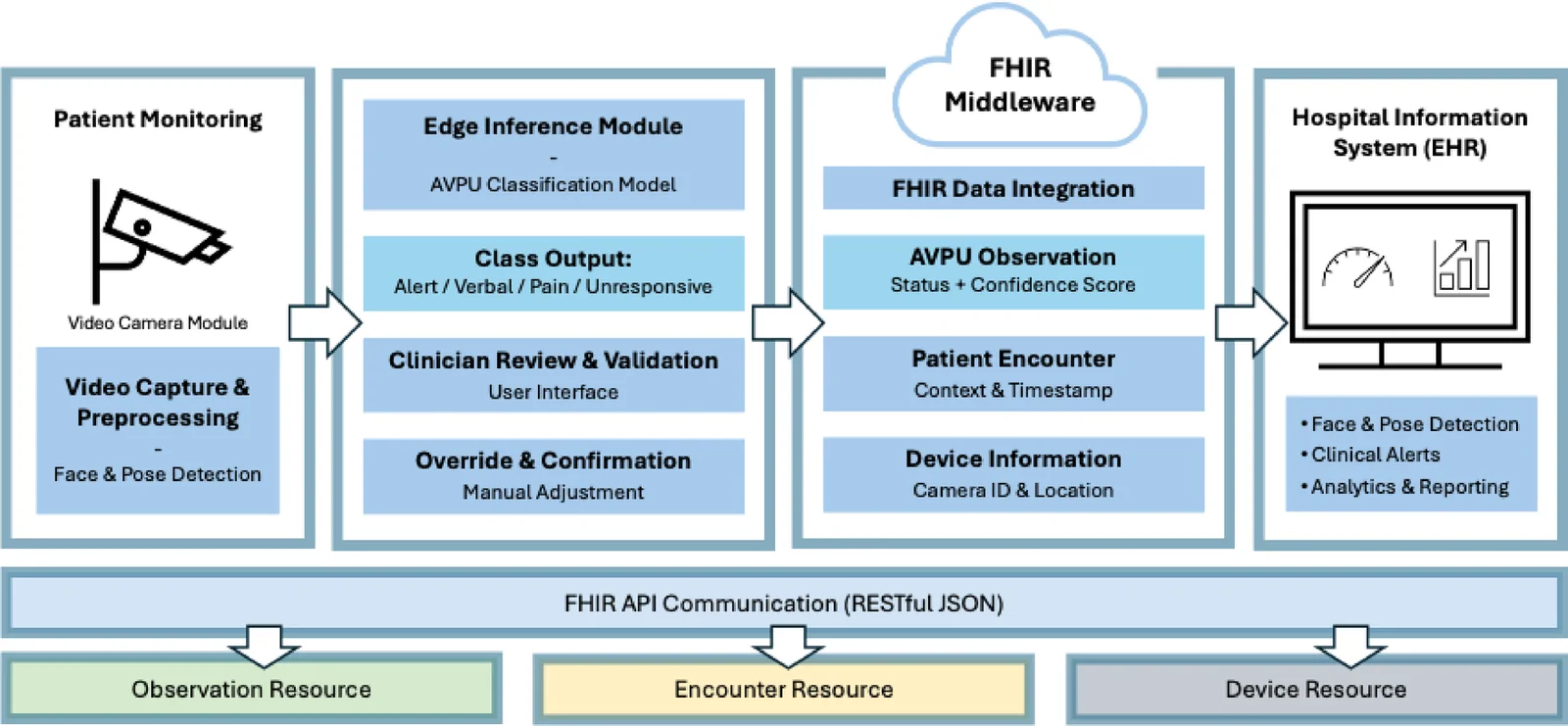
AVPU video-based consciousness detection system with FHIR event flow integration.

The objective of the proposed system is to produce a reliable, automated AVPU classification that can be used across Emergency Medical Services (EMS), Emergency Departments (ED), and Intermediate Care (IC) wards. The conceptual framework is built around three principles: (1) the staff remains responsible for the AVPU procedure; (2) the system performs inference on the patient’s responsiveness using video-based computer vision; and (3) the inferred AVPU classification is seamlessly integrated into clinical information systems and triage workflows (with staff validation), see Fig. 1.

The staff initiates a standard AVPU assessment, involving verbal prompts or application of stimuli as clinically appropriate. A camera captures the patient’s facial and behavioural responses in real time (upper torso and heads, face/pose detection). The video stream is processed either locally (edge computing) or on a secure institutional server, depending on the scenario. The inference model outputs a predicted AVPU category with a corresponding confidence score. The clinician can validate, accept, or override the result before it is registered in triage software, EMS platforms, or electronic health records (EHR). The event flow is represented as FHIR resources—primarily Observation (AVPU state + confidence + timestamp), linked to the relevant Encounter (care context) and Device (camera identifier/location)—and then propagated to the hospital EHR for documentation, alerting, and analytics.

This semi-automated workflow preserves clinician oversight while providing objective, consistent assessments, mitigating documentation gaps and reducing dependence on subjective interpretation.

The system uses a static or mobile camera with sufficient resolution and lighting dynamic range (preferably 1080p or above) and stable frame rate to capture subtle facial expressions and purposeful movements. Camera placement varies by scenario: mounted on an ambulance ceiling rail, fixed on a triage desk, or installed above a ward bed. Dedicated lighting is recommended to ensure consistent visual conditions.

**Fig. 2:**
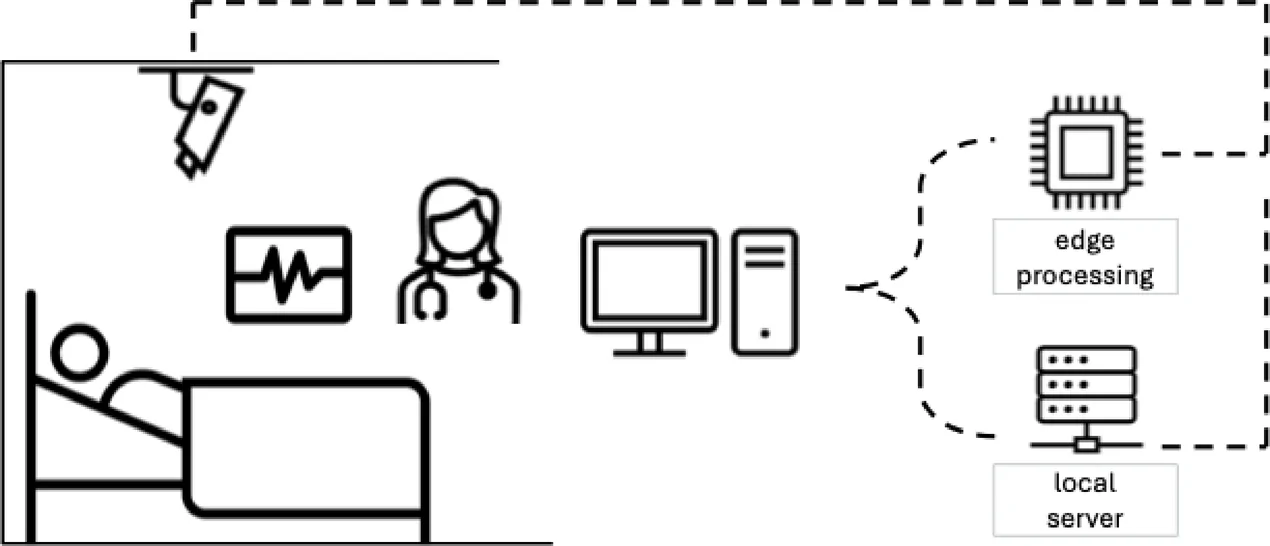
Experimental setup for video data collection in the AVPU consciousness detection study.

Medical-grade cameras may be considered in demanding environments (example: operating room due to sterilisation procedures). Non-contact video systems may reduce some hardware regulatory constraints facilitating integration in multiple clinical contexts, such as wards, if they are not in itself a medical diagnostic tool or a decision-support tool, but software classification and clinical evidence obligations may remain according to MDCG 2019-11^31^, see Fig. 2.

Processing Unit (Local or Remote). The processing unit is responsible for running the machine learning model. Two architectures are possible:

1. Local edge computing: Inference occurs directly at the point of care using embedded GPU devices or compact inference hardware. This configuration ensures low latency, high privacy, and independence from network interruptions;
2. Remote processing: The video stream is transmitted to a secure hospital server for inference. This configuration allows heavier models and supports centralised maintenance.

The choice depends on each scenario’s technical constraints and network reliability. Local processing is much preferable because of privacy concerns.

AVPU Classification Model. The model interprets facial landmarks, eye opening, head orientation, and motor responses associated with verbal or painful stimuli and the classifier maps the extracted features to one of the four AVPU categories. Each video stream is processed frame-by-frame to detect and localize the patient’s face, after which computer vision algorithms extract pose/facial landmark features representing motion and responsiveness cues. These per-frame features are aggregated through temporal windowing to encode short-term dynamics (e.g., reaction latency and movement patterns) into fixed-length sequences. The resulting structured representation is formatted as a model input tensor, which is then used by the AVPU classifier to infer the patient’s responsiveness state. Confidence scores accompany each prediction to support clinical validation^30^.

**Fig. 3:**
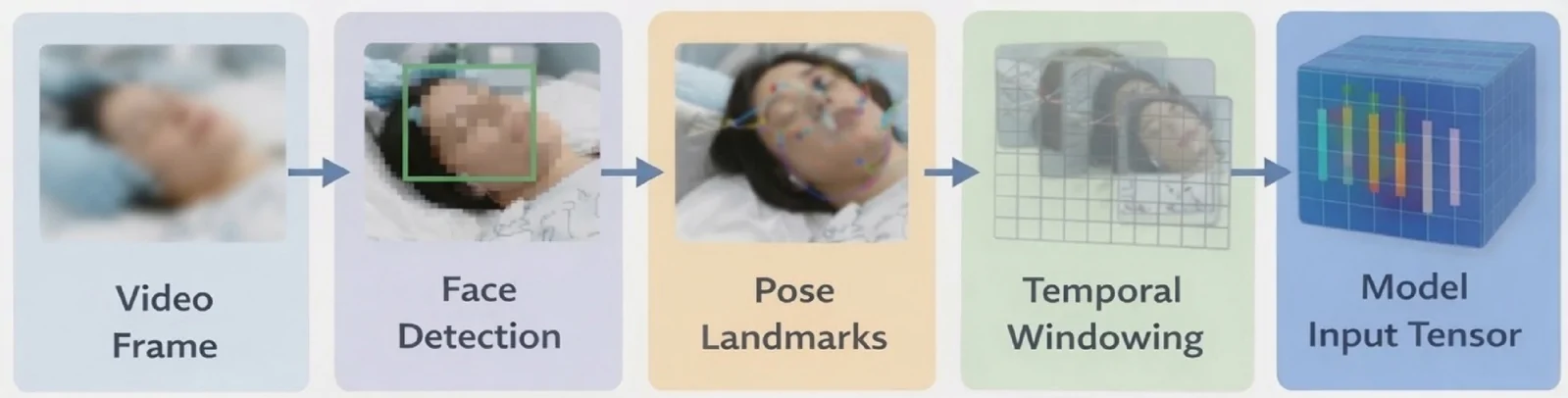
Feature extraction pipeline for AVPU video-based consciousness classification.

Depending on the clinical context, the model may analyse short video windows or continuous streaming until the assessment concludes. In Fig. 3, each video frame is first captured from the recorded sequence and processed using a face detection algorithm to localize the patient’s face. Facial pose landmarks are then extracted to represent key spatial features of facial movements and expressions. These landmark sequences are organized into temporal windows to capture motion dynamics across consecutive frames. Finally, the resulting structured data are converted into a model input tensor, which is used by the deep learning model to perform temporal classification of patient responses.

Integration Middleware. This layer handles the secure transmission of AVPU outputs, timestamps, and metadata to existing clinical systems, enhancing traceability. It implements standard communication protocols such as RESTful APIs or FHIR-compliant message structures. The middleware supports: (1) EHR integration; (2) EMS–hospital handover exchanges; (3) NEWS score calculators; and (4) Ward monitoring dashboards.

It also ensures authenticated, encrypted communication and proper mapping to patient identifiers and clinical encounters.

User Interface for Validation. A minimal interface presents the AVPU prediction and confidence level to the clinician. The user can confirm or override the result and provide an optional comment. This step is essential for clinical accountability and ensures that the system functions as a decision-support tool rather than a replacement for clinical judgement.

Integration with Clinical Pathways. EMS settings require rapid, stable, and autonomous operation. The architecture supports the complete AVPU assessment within the ambulance:

1. Technician activates the AVPU assessment mode;
2. The camera records the interaction;
3. The local processor generates the AVPU score;
4. The technician validates the result;
5. The score is added to the EMS software and included in the electronic patient handover;
6. Relevant data are communicated to the EMS coordination centre and the receiving hospital.

This process enhances handover accuracy by ensuring consistent AVPU data, which often plays a major role in destination decisions and risk communication.

Emergency Department Triage. In the ED, AVPU classification occurs during triage performed by a nurse or technical staff. The proposed architecture integrates with existing triage software or the hospital’s EHR interface:

1. The triage officer initiates the AVPU assessment;
2. The camera and local processor generate the classification;
3. The officer validates the output and completes the remaining physiological measurements;
4. The AVPU value is automatically integrated into the NEWS computation and triage category;
5. The complete triage record is uploaded to the EHR.

Automating AVPU aims to reduce inter-rater variability and ensures that mental status is reliably captured during initial assessment.

Intermediate Care Wards. In intermediate care, patients are intermittently monitored for some vital parameters (contrasting to the ICU). Due to the lower criticality of the patients, the staffing ratios determinate less frequent observations. Also, the ward layout does not provide a direct line of sight between the nurses offices and ward beds, limiting any informal patient assessment just based on eyesight. The architecture supports both on-demand and scheduled assessments:

1. The nurse initiates an AVPU assessment at predefined intervals;
2. The ceiling-mounted camera captures facial and behavioural responses;
3. The processor computes the AVPU value;
4. The nurse confirms the result and documents any contextual notes;
5. The ward monitoring system forwards the result to the EHR.

This model reduces missed detections of evolving AMS, supporting earlier detection of delirium, sepsis, or neurological decline, by encouraging staff routine supported by automated workflows.

Harmonisation Across Environments. To ensure continuity of care, AVPU assessments from EMS, ED, and wards are recorded with unified data structures and timestamps. When combined with NEWS and other physiological parameters, this enables longitudinal clinical interpretation from pre-hospital to inpatient phases.

### Data standardisation and interoperability using FHIR

FHIR-Based Representation of AVPU. To integrate the AVPU result with modern clinical information systems, the output is mapped to HL7 FHIR structures. The AVPU score is represented as an Observation resource that includes:

- Coded value representing A, V, P, or U (mapped to SNOMED CT: 271594007 – Alert, 260544009 – Responds to voice, 260546006 – Responds to pain, 260545005 – Unresponsive; with Observation.code set to LOINC 67775-7 – “Level of responsiveness”, under FHIR R4);
- Timestamp of the assessment;
- Performer (staff identifier);
- Device reference (camera or processing unit);
- Confidence value from the model;
- Link to the Encounter resource (EMS call, ED visit, or ward encounter).

Using FHIR ensures compatibility with diverse healthcare platforms and supports the exchange of structured triage data across EMS and hospital systems.

From an implementation standpoint, the integration middleware exposes a RESTful FHIR R4 endpoint (POST /Observation) to which the inference engine submits each validated assessment. The Observation.status field is set to “final” only after clinician confirmation; prior to that it carries “preliminary”, preserving the provenance of machine-generated versus clinician-confirmed values. The Observation.encounter links to the active EMS or hospital Encounter, enabling downstream NEWS calculators to retrieve consciousness data via standard FHIR search parameters (e.g., GET /Observation? patient = < id>&code = 67775-7). Confidence scores are stored as a supplemental Observation.component.valueDecimal element, providing decision-support transparency without altering the primary coded value.

Integration with NEWS. AVPU values are forwarded to the NEWS calculator within the triage or ward software. Because the NEWS scoring matrix treats V, P, and U states as critical deviations from baseline, accurate and consistent AVPU documentation is essential for:

- Detecting deterioration;
- Activating rapid response teams;
- Identifying sepsis risk;
- Determining transfer to higher acuity units.

The integration architecture ensures that AVPU information is systematically included in NEWS computations, reducing human omission.

Operational Validation Flow. The operational validation flow is designed to align with clinical governance and safety requirements.

1. Initiation: Clinician starts AVPU assessment; system records video;
2. Inference: Local or remote processing generates AVPU prediction with confidence score;
3. Validation: Clinician reviews the output, confirms or overrides the result;
4. Registration: Validated AVPU score is inserted into the triage or EHR system;
5. Integration: NEWS score updated when appropriate; data forwarded to relevant clinical dashboards;
6. Storage: Only numerical and metadata outputs are stored; raw video is not retained unless explicitly permitted by policy;
7. Audit: System logs timestamps, user actions, and device identifiers for accountability.

This flow ensures that the system remains a support tool and that clinicians retain full control over final clinical documentation.

Safety by Design. Although this thesis does not include the mandatory MDR (Medical Device Regulation) pathway for certification, required for the commercial stage of the product, it is important to include in the architecture concept design features for the foundations of a future safe and fully certified product. “Safety by Design” is a term applied to several areas of product and service development and use, either physical or digital systems that aim to provide guidance while incorporating, assessing and enhancing user safety. Some aspects should be considered for this specific development as best practice, namely Safety by Design principles^32^.

Under MDCG 2019-11^31^ guidance, Consc.ia would qualify as Software as a Medical Device (SaMD). Because the system informs rather than autonomously executes clinical decisions, its initial risk classification under Rule 11 of MDR Annex VIII would likely be Class IIa, requiring conformity assessment by an EU Notified Body, ISO 13,485 quality management certification, and a clinical evaluation report under Article 61 of Regulation (EU) 2017/745. Medico-legal responsibility for the final documented AVPU value rests with the confirming clinician, consistent with the clinician-in-the-loop design principle. Any prospective pathway to market requires: (i) a Technical Documentation file, (ii) post-market clinical follow-up, and (iii) registration in EUDAMED. These obligations are acknowledged as explicit milestones on the product roadmap.

- Service provider responsibilities: the burden of safety should never fall solely upon the end user. Service providers can take preventative steps to ensure that their service is less likely to facilitate, inflame or encourage illegal and inappropriate behaviours;
- User empowerment and autonomy: the dignity and safety of users is of central importance, with users’ best interests a primary consideration;
- Transparency and accountability: transparency and accountability are hallmarks of a robust approach to safety. They not only provide assurances that services are operating according to their published safety objectives but also assist in educating and empowering users about steps they can take to address safety concerns;
- Safe configurations by default: predefined default configurations should be the ones the product or service ensures the higher degree of protection or privacy.

Additionally, another remark must be made because the product contextualised in the thesis is driven by ML algorithms running on a video feed. Although machine learning has advanced rapidly in healthcare, a critical missing piece in most models is the ability to quantify and communicate uncertainty for individual predictions^33^. Current ML/AI systems typically provide a point estimate (a single prediction) without expressing how confident the model is, which in clinical settings can mislead decision-making or lead to unsafe outcomes.

In healthcare, a wrong or overconfident prediction can lead to incorrect diagnoses or treatment choices. If clinicians can understand when a model is uncertain, they can escalate to human expertise, seek more data, or order additional tests rather than act on a potentially unreliable output. Thus, ML/AI models should behave similarly to human experts in clinical reasoning, opting for asking for a second opinion or additional information in the face of uncertainty. Over time this feature of uncertainty enhances both safety and trust in the system, and future good practices point that uncertainty quantification should become a standard part of ML/AI evaluation and reporting products and services.

## “Consc.ia” evaluation and implementation

While the proposed architecture for automated AVPU classification is conceptually uniform, its operationalisation must be adapted to the specific characteristics of each clinical environment. Emergency Medical Services, Emergency Departments, and Intermediate Care wards differ significantly in terms of workflow, physical layout, staffing, network availability, and integration requirements. This chapter describes three concrete implementation scenarios, illustrating how the system can be configured and deployed within each setting. The intention is not only to demonstrate technical feasibility, but also to highlight the practical considerations necessary for real-world adoption.

Scenario 1 — Emergency Medical Services (EMS). Operational Context. EMS personnel often perform AVPU assessments under time pressure and in dynamic, constrained environments. Ambulances vary in layout, lighting, and available space. Connectivity may be intermittent during transport, making autonomous local processing essential. The AVPU assessment is typically performed during initial patient contact and monitored intermittently during transport. Accurate consciousness documentation plays a crucial role in:

- Determining transport priority;
- Influencing the choice of destination hospital;
- Communicating patient severity to coordination centres;
- Supporting timely activation of hospital teams.

**Fig. 4:**
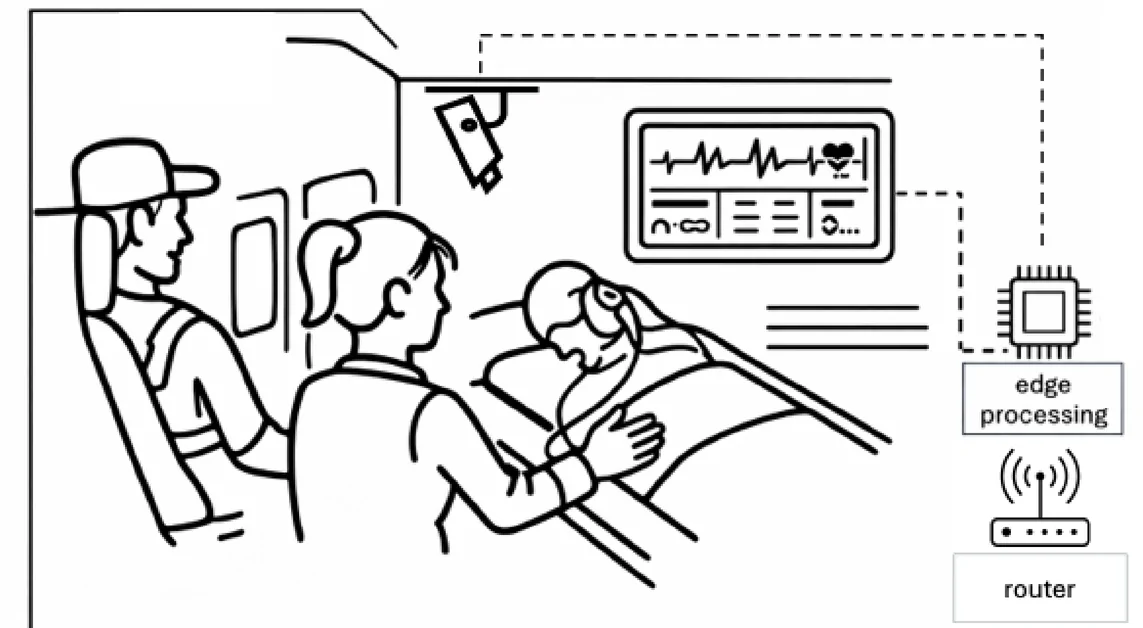
EMS Scenario concept, inside an ambulance, partially AI-generated.

The EMS setup includes: (1) a fixed camera mounted on the ambulance ceiling or wall, aimed at the stretcher; (2) integrated or auxiliary LED lighting for consistent illumination; (3) An edge processor installed within the ambulance equipment area and (4) Optional battery backup to ensure uninterrupted operation, see Fig. 4.

The camera should ideally offer at least 1080p resolution, a wide dynamic range, and autofocus. Medical-grade certification is generally not required since the system does not physically contact the patient.

The EMS workflow proceeds as follows: (1) The technician initiates the AVPU assessment using the EMS software; (2) The camera captures the patient’s facial responses as verbal or painful stimuli are applied; (3) The edge processor performs real-time inference and produces an AVPU classification; (4) The technician validates the result and confirms or overrides it; (5) The result is automatically recorded in the EMS system; (6) Consciousness level and associated NEWS data are transmitted to coordination centres; and (7) Upon arrival at the hospital, the validated AVPU result is included in the electronic handover.

Technical Constraints and Expected Benefits: The EMS environment introduces specific technical challenges: (1) Variability in patient positioning on the stretcher; (2) Vibration and movement during transport; (3) Potential camera occlusion by clinicians; (4) Limited and inconsistent lighting; (5) Inconsistent connectivity; and (6) Safety requirements for mounted equipment in moving vehicles.

Edge processing ensures that inference remains functional even without network access. Only numerical results—not raw video—need to be transmitted to preserve privacy and minimize bandwidth use.

These are the expected benefits for this scenario: (1) Reduction in incomplete EMS records; (2) More reliable handovers due to objective consciousness assessment; (3) Support for early detection of deterioration during transport; (4) Improved coordination between EMS and hospital staff; and (5) Potentially drive health outcomes.

Scenario 2 — Emergency Department (ED). Operational Context.

ED triage is structured, high-throughput, and time-sensitive. Assessments are typically performed by trained triage nurses using the Manchester Triage System (MTS) combined with NEWS/NEWS2 scoring. Within this environment, the AVPU score influences triage category, risk stratification, and resource allocation. An automated system must fit seamlessly within the triage workflow without increasing workload or lengthening assessment time.

**Fig. 5:**
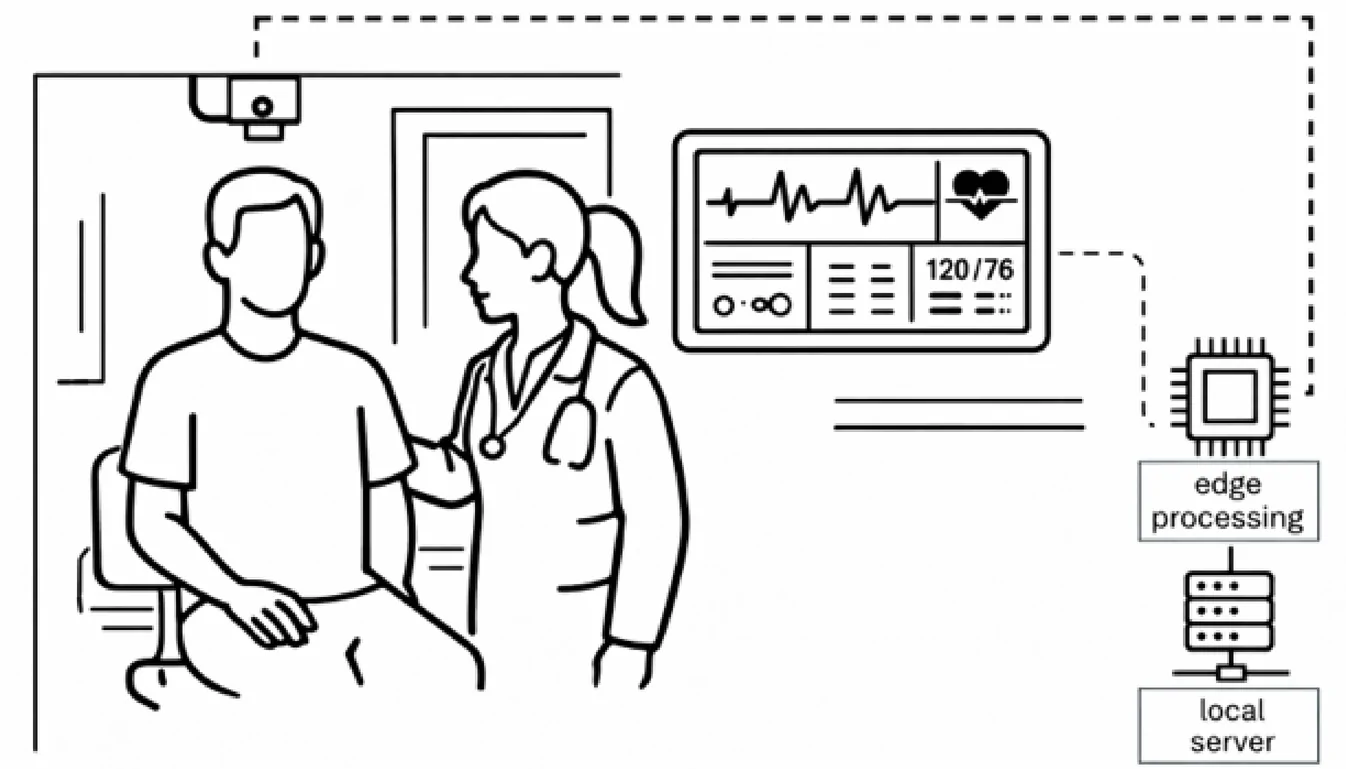
ED Scenario concept, a triage room, partially AI-generated.

The ED implementation uses, see Fig. 5: (1) a camera fixed to the triage station or mounted facing the patient; (2) a stable lighting environment, usually already present in triage rooms; (3) either a desktop inference unit or a shared hospital server and (4) Direct network connection to the hospital’s internal LAN.

The camera placement ensures unobstructed capture of the patient’s face during triage questioning and stimulation.

The ED triage process follows these steps: (1) The triage nurse initiates the AVPU module within the triage software; (2) The system captures the patient’s response as part of routine questioning; (3) Inference is performed locally or via a hospital server; (4) The nurse reviews the classification result and validates it; (5) The AVPU value populates the required field in both the MTS and NEWS assessment forms; and (6) The completed triage record is stored in the EHR.

Because ED environments are more controlled than EMS settings, inference reliability is higher and integration with EHR systems is more straightforward.

These are the technical constraints for this scenario: (1) Integration with MTS software; (2) Secure connection to EHR systems; (3) Storage policies for protected health information; and (4) Need to ensure minimal assessment delays.

These are the expected benefits for this scenario: (1) Consistent AVPU documentation, reducing missing data; (2) Improved triage reliability and reproducibility; (3) Enhanced NEWS accuracy; (4) Reduction in inter-rater variability between triage professionals; and (5) Potentially drive health outcomes.

Scenario 3 — Intermediate Care (IC) Wards. Operational Context.

Intermediate Care wards provide monitoring for patients at risk of deterioration but not requiring full ICU support. Staffing ratios typically range from one nurse per four to six patients, meaning that continuous observation is not always feasible. Consciousness level is recorded at scheduled intervals, often with significant delay between assessments. The architectural layout often prevents direct visibility from the nurse station to all beds.

The proposed system addresses these limitations by enabling both scheduled and on-demand AVPU assessments with minimal effort from staff.

In the IC ward, the system consists of: (1) ceiling-mounted cameras oriented toward each patient bed; (2) controlled lighting or infrared illumination to reduce environmental variability; (3) a ward-based edge server capable of processing multiple video streams and (4) integration with ward monitoring dashboards.

Because the hardware operates continuously, energy efficiency and thermal stability are important considerations.

The IC workflow operates in two modes: Scheduled assessments (1) At predetermined times, the nurse initiates an AVPU evaluation at the bedside. (2) The camera and processor capture and interpret the patient’s responsiveness. (3) The nurse validates the classification. (4) The AVPU value is added to the ward monitoring record and EHR.

On-demand assessments (1) The nurse or monitoring system triggers an additional AVPU assessment due to clinical suspicion. (2) The system conducts the evaluation as in scheduled mode.

These assessments complement continuous vital-sign monitoring, supporting early detection of clinical deterioration such as delirium, hypoxia, sepsis, or neurological decline.

These are the technical constraints for this scenario: (1) Need for multi-camera management and synchronised inference; (2) Variable patient orientation; (3) Risk of partial occlusion due to blankets or bed rails; and (4) Potential need for thermal or low-light camera options.

These are the expected benefits for this scenario: (1) Reduction in missed changes in mental status; (2) Earlier recognition of deterioration and reduced time-to-escalation; (3) Enhanced patient safety; (4) Continuous documentation without additional workload; (5) Improved accuracy of ward-level NEWS scoring; and (6) Potentially drive health outcomes.

Comparative Analysis Across Scenarios. Several metrics and resources should be considered for a cross-scenario comparison. Regarding latency and processing requirements:

- EMS requires low-latency edge processing due to variable connectivity;
- ED allows either local or centralised processing;
- IC wards require multi-stream processing and scalability.

Regarding workflow considerations:

- EMS workflows are the most time-pressured, requiring rapid confirmation;
- ED workflows are structured and benefit from improved standardisation;
- IC wards gain the most from supplemental functionality due to limited staff visibility.

Regarding IT integration:

- EMS systems typically rely on proprietary platforms; integration requires dedicated APIs;
- ED systems are well-integrated with hospital EHRs, facilitating FHIR-based exchanges;
- IC wards may have additional nursing documentation systems between bedside and EHR layers.

On the theme of resources required:

- EMS: single camera and portable processor;
- ED: fixed camera and workstation/server;
- IC: multi-camera arrays and ward-level processors.

And finally, on clinical impact:

- EMS: improves pre-hospital risk communication and destination planning;
- ED: optimizes triage accuracy and reduces human omission;
- IC: supports early intervention and reduces unnoticed deterioration.

### Stakeholder validation and dataset development

Adopting a product- and market-oriented approach, the development of Consc.ia included a continuous evaluation process based on (i) stakeholder engagement and (ii) dataset development. The objective of this process was to validate the existence of a real operational need, refine product assumptions (usability and workflow fit), and identify constraints related to IT integration and data privacy.

The evaluation activities were designed to address the following aspects:

- Market and clinical need validation: confirm the relevance of AVPU documentation challenges across EMS and hospital contexts.
- Usability and user experience inputs: collect feedback on interface expectations and user-friendly interaction under time pressure.
- Workflow integration feasibility: understand where and how the solution could be embedded in EMS/ED routines and decision-making processes.
- IT and interoperability constraints: identify integration expectations with existing systems and documentation practices.
- Privacy and governance concerns: understand operational acceptance conditions regarding video capture, retention, and consent.

Healthcare services—both EMS and hospital settings—operate under severe constraints in time and personnel. Early-stage technology validation is further limited by organisational hierarchy, access controls, and ethics and governance requirements. For these reasons, and to enable timely iteration during the thesis timeframe, interviews and recordings were primarily obtained through professional networks and informal recruitment channels. This approach facilitated access to real-world users and realistic contexts for simulated AVPU assessment procedures, while remaining compliant with ethics and privacy requirements.

### Dataset profiling

The dataset used in the proof-of-concept work comprises the following characteristics:

- Total number of videos: 136.
- Number of distinct participants: 58.
- Recruitment environments: ISCTE University, hospital setting, EMS unit, fire department.
- Gender: 36 female, 22 male.
- Age distribution: approximately 90% between 25 and 55 years old.
- Professional background: physicians (6), nurses (30), paramedics (6), first responders (10), healthcare consultants (6).
- Simulated AVPU procedures included: verbal stimulation, pain stimulation, and unresponsive scenarios (136 independent recordings).
- Recording batches (date and number of entries):

**Table:** 

2025-03-16 (7)	2025-04-26 (12)	2025-04-17 (24)	2025-04-25 (16)
2025-04-26 (12)	2025-05-05 (8)	2025-05-06 (6)	2025-05-08 (47)

### Ethics, consent, and privacy safeguards

Data collection and privacy protection were formally addressed through institutional governance. ISCTE’s ethics commission issued a positive opinion for the study and defined procedures to protect participants’ privacy (ref. CE-ISTA/2025.08). All participants received a participant information sheet and signed an informed consent form prior to recording. In line with privacy-by-design principles, the evaluation focused on feasibility and documentation workflows, minimising unnecessary retention and exposure of raw video data.

### Stakeholder feedback through ManagiDiTH summer week

During the ManagiDiTH Summer Week held at ISCTE Lisboa (May 2025), the author participated as a representative of an external business entity and proposed a business-oriented healthcare challenge aligned with the dissertation topic. The challenge focused on the medical relevance of consciousness assessment (AVPU/CL), the observed gaps in routine scoring practices, and the potential role of computer vision as an enabling approach.

A five-minute pitch was delivered to an audience of approximately 200 participants, including healthcare professionals (physicians, nurses, and IT staff) and members of the ManagiDiTH academic community. The challenge was subsequently explored by a student group in an Innovation Bootcamp, supported by a written brief provided in the annexe 3. Feedback from attendees working in operational healthcare contexts reinforced the perceived relevance of the problem and the potential value of a solution that supports consistent documentation.

Overall, stakeholder engagement activities (interviews and Summer Week feedback) provided consistent indications of (i) the relevance of AVPU documentation quality, (ii) the importance of workflow-friendly interaction, (iii) the need for interoperable documentation and minimal disruption to existing systems, and (iv) strong privacy expectations regarding video handling.

A key limitation is that in-situ proof-of-concept testing using dedicated hardware in live healthcare settings could not be conducted within the thesis timeframe due to operational constraints and access limitations. As a result, this thesis positions Consc.ia as a proof-of-concept prototype supported by stakeholder validation and controlled/simulated data collection, establishing a foundation for subsequent pilots, clinical validation studies, and product maturation, see Table 2.

**Table 2:** Summary of Evaluation Methods.

Purpose	Participants / sample	Outputs produced	Limitations
Evaluation method: Stakeholder interviews and informal contacts			
Validate operational need; gather requirements for usability, workflow fit, IT integration, and privacy constraints	Healthcare professionals engaged via professional networks across EMS and hospital contexts (roles represented in the dataset profile: physicians, nurses, paramedics, first responders, consultants)	Consolidated qualitative insights on: expected UI simplicity, clinician-in-the-loop validation requirement, workflow integration points, interoperability expectations, and privacy-by-design constraints	Sampling driven by accessibility; limited time availability of clinicians; insights are qualitative and may not represent all institutions; no controlled usability study conducted
Evaluation method: Workshop pitch (ManagiDiTH Summer Week, May 2025)			
Rapid market/need sensing; test clarity and resonance of the problem statement and solution premise	~ 200 attendees including healthcare professionals (doctors, nurses, IT staff) and academic participants	Audience-level validation that the AVPU/CL documentation gap is recognised; confirmation of perceived relevance and interest in a computer-vision-supported approach	Feedback is not a structured evaluation instrument; self-selection bias (attendees interested in innovation); no systematic measurement of acceptance or usability
Evaluation method: Innovation Bootcamp challenge (student group work)			
Stress-test the problem framing and explore solution assumptions from a practical and implementation-oriented perspective	Student team(s) participating in the Innovation Bootcamp, using the provided brief (Annexe 1)	Refined articulation of requirements and constraints; additional perspectives on feasibility, integration assumptions, and potential product features	Participants are not end-users; outputs are exploratory; not equivalent to clinical validation or operational deployment evidence
Evaluation method: Dataset collection (simulated AVPU procedures)			
Enable proof-of-concept development and feasibility evaluation; collect realistic interaction patterns for AVPU simulations	136 videos; 58 distinct participants; recruited from university, hospital, EMS unit, fire department; roles include MD, nurse, paramedic, first responder, consultant	Dataset enabling PoC modelling and system testing; structured batch tracking; informed consent and ethics approval (CE-ISTA/2025.08)	Simulated procedures rather than real patients; potential domain shift; limited age distribution; environment variability not fully controlled; in-situ pilot hardware validation not performed in live care settings
Purpose	Participants / sample	Outputs produced	Limitations

This chapter demonstrated how the proposed architecture for automated AVPU assessment can be adapted to three distinct clinical contexts. EMS, ED, and Intermediate Care environments differ substantially in workflow, physical conditions, staffing, and information systems. Yet in all cases, automated AVPU classification addresses a persistent gap in documentation and risk detection. These implementation scenarios illustrate practical pathways for deploying the system progressively across the continuum of emergency care. Stakeholder involvement is precious along product development continuum and benefits the final product adoption.

## Discussion

This chapter synthesizes the clinical, technological, and operational implications of implementing an automated AVPU classification system across Emergency Medical Services (EMS), Emergency Departments (ED), and Intermediate Care (IC) wards. The goal is to interpret the findings from previous chapters, evaluate the potential benefits and limitations of the proposed approach, and discuss its broader implications for patient safety, digital transformation, and workflow optimisation. This chapter also considers ethical, legal, and implementation challenges, and identifies directions for future development.

### Clinical Impact

Improving Reliability of Consciousness Assessment. Consciousness level is a critical determinant within triage systems such as NEWS. However, AVPU documentation is often incomplete or subjective, especially in fast-paced settings. Automated AVPU classification offers a standardised and reproducible method of assessing responsiveness, reducing reliance on subjective interpretation.

The system enhances clinical reliability by:

- Reducing missed or unrecorded AVPU values, improving NEWS score;
- Providing consistent evaluations across staff, departments and services;
- Enabling early detection of changes in consciousness during transport or ward care;
- Supporting rapid response activation when consciousness deteriorates.

This has the potential to improve patient outcomes, particularly in cases were evolving altered mental status signals acute deterioration.

Enhancing Continuity Across Care Pathways. The integration of AVPU results across EMS, ED, and IC environments creates a continuous record of consciousness evaluation from pre-hospital contact to inpatient care. This continuity strengthens clinical handovers, supports better risk communication, and ensures that early warning systems remain accurate throughout the patient journey.

Operational Benefits. Reducing Documentation Burden. Manual AVPU documentation contributes to cognitive load and can be easily omitted during high-pressure situations. The proposed system automates the capture and recording of consciousness level, requiring only clinician validation. This reduces administrative tasks while maintaining data quality.

Supporting Workforce Efficiency. In IC wards, where staffing ratios limit direct observation, automated AVPU assessments offer supplemental monitoring without increasing nurse workload. In EMS, where time is constrained and environments are unstable, automation supports efficient and standardised assessment.

Enhancing Training and Consistency. AVPU assessment is simple but can still exhibit variability across providers. The automated system may serve as a reference point for less experienced staff, encouraging greater adherence to standardised assessment protocols.

### Technical feasibility and constraints

Real-Time Performance. Edge computing enables rapid inference without reliance on network connectivity, particularly important in ambulances and triage areas. Modern hardware platforms support inference at low latency, making real-time AVPU classification feasible within current technological limits.

Environmental Challenges. Despite feasibility, the system faces several environmental constraints: (1) Inconsistent lighting; (2) Patient occlusion; (3) Variable positioning of patients; (4) Movement or agitation; and (5) Background noise and personnel traffic.

These challenges require robust model training and environmental calibration. Scenario-specific design guidelines, as outlined in Chap. 5, are crucial to ensuring reliable operation.

Input Video Quality. A cross-cutting concern for all deployment scenarios is the quality of the incoming video stream. The performance of any computer vision pipeline is fundamentally bounded by image and video quality; artefacts such as motion blur, compression noise, low illumination, or sensor noise can substantially degrade feature-extraction accuracy. In clinical environments, video quality is further affected by camera-to-patient distance, patient movement, and dynamic lighting variability. Research on blind image quality estimation demonstrates that no-reference quality metrics can reliably characterise these artefacts without a pristine reference frame^34,35^, and work on subjective and objective quality assessment of audiovisual signals shows that human perceptual ratings and automated metrics diverge in ways that matter for AI-driven pipelines^36,37^. These insights motivate including a real-time quality monitoring module in the Consc.ia middleware: frames falling below a minimum quality threshold should trigger a pause of inference and an alert to the clinician, preventing low-confidence predictions from degraded input and preserving patient safety.

Integration with Existing Information Systems. Integration into EHR platforms, EMS systems, and triage software is essential. FHIR-based interoperability enables such integration, but real-world implementations may still require custom APIs and vendor collaboration.

### Ethical, legal, and privacy considerations

**Fig. 6:**
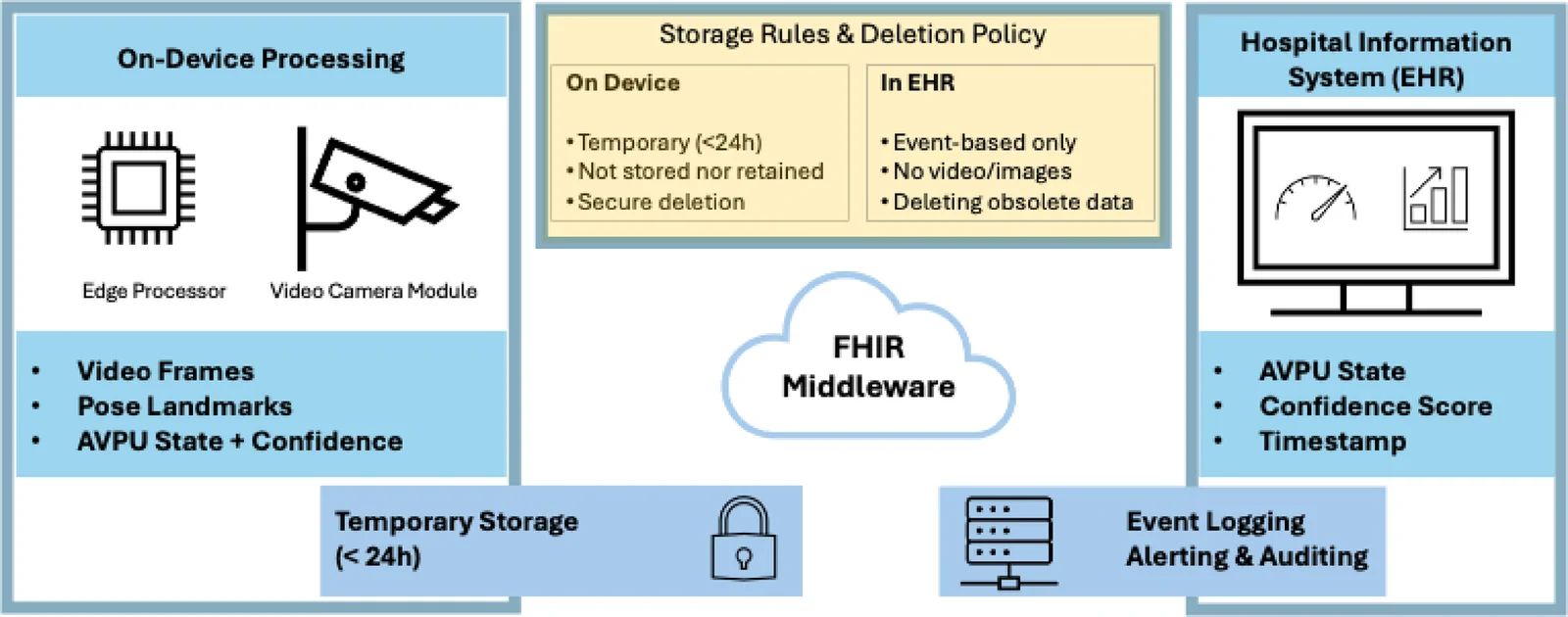
Video capturing of AVPU scoring and EHR register, with minimum patient information retention, draft design.

Video Capture in Clinical Environments, see Fig. 6. Recording patients introduces significant ethical and legal concerns, particularly under stringent privacy regulations such as GDPR. Key safeguards include:

- Processing video locally to avoid transmission of raw footage;
- Avoiding storage of video unless explicitly required and consented;
- Minimising captured areas to only the patient’s face;
- Encrypting all data in transit and at rest;
- Implementing strict role-based access controls.

By designing the system for local inference and ephemeral video handling, privacy risks can be substantially reduced.

Clinical Responsibility and Oversight. The automated AVPU classification system functions as a decision-support tool, not a diagnostic or autonomous decision-maker. Clinician validation remains central to: (1) Ensuring medical appropriateness; (2) Preventing misinterpretation; and (3) Maintaining medico-legal accountability.

The system must be transparent about its limitations and avoid creating a false sense of certainty.

### Bias and fairness

Computer vision models may exhibit performance differences across demographic groups if training data are unbalanced. Consciousness assessment, which may rely on facial features or responsiveness, requires careful evaluation across: (1) Skin tones; (2) Age groups; (3) Facial morphologies; and (4) Clinical pathologies and therapeutics (which can interfere).

Ensuring fairness and minimising bias requires diverse training data and ongoing validation studies.

### Implementation challenges

Clinical environments are resistant to changes that introduce additional complexity. Although the proposed system minimizes workflow disruption, acceptance depends on: (1) Perceived usefulness; (2) Ease of use; (3) Training quality; and (4) Support from clinical leaders.

Pilot studies with iterative feedback loops are essential for successful adoption.

Technical integration varies among hospitals. Some institutions have mature digital ecosystems, while others have fragmented systems. Deployment requires: (1) Stable local networks; (2) Compatible EHR platforms; (3) Support from IT departments; and (4) Maintenance plans for hardware and software.

Costs related to hardware acquisition, integration, maintenance, and training must be justified by clinical benefits, operational efficiencies, or reduced adverse events. A full cost-benefit analysis, while outside the scope of this thesis, is recommended during pilot phases.

The proposed architecture for automated AVPU classification offers meaningful improvements to triage reliability, clinical communication, and early detection of deterioration. While technically feasible and clinically valuable, successful adoption requires careful attention to workflow integration, privacy safeguards, staff acceptance, and interoperability. The diffusion modelling presented in Chap. 6 demonstrates that adoption will likely be gradual, influenced by demonstrable value, regulatory guidance, and institutional readiness.

The next chapter concludes the dissertation by summarising the core contributions and outlining the path forward for clinical deployment and research.

## Conclusion

This research work examined the feasibility, architecture, and potential adoption of an automated video-based AVPU classification prototype system designated by Consc.ia, intended for deployment across Emergency Medical Services, Emergency Departments, and Intermediate Care wards. The work addressed clinical, operational, and technological gaps associated with consciousness assessment and demonstrated how computer vision and edge computing can support more consistent and reliable documentation within early warning and triage systems.

AVPU assessment, despite its simplicity and clinical relevance, remains one of the most inconsistently recorded components of triage workflows. Its omission significantly affects NEWS scoring accuracy, continuity of care, and early detection of patient deterioration. This dissertation proposes a structured solution that automates AVPU evaluation while preserving clinician oversight, ensuring interoperability with health information systems, and supporting integration into diverse care environments, maintaining a high robustness of data privacy and ethical concerns.

Product adoption scenarios were explored identifying stakeholders, market variables and segments, and provided a set of working parameters for roadmap monitoring and business steering, adapting to scale and market maturity.

This work contributes to the domains of emergency medicine, digital health, and clinical decision-support systems in several ways.

First, it clarifies the clinical significance of consciousness assessment within triage systems and highlights the widespread problem of missing or inconsistent AVPU documentation. The analysis demonstrates that this gap has direct implications for patient safety and risk stratification.

Second, it investigates the technological foundations necessary for automated AVPU assessment, including camera-based behavioural analysis, real-time computer vision models, and edge computing architectures. These components establish a feasible basis for implementing real-time automated consciousness evaluation, integrated in Consc.ia prototype design.

Third, the dissertation introduces a comprehensive architecture for an AVPU classification system. The framework integrates hardware, inference pipelines, validation interfaces, interoperability layers, and workflow design, ensuring the system can be deployed across EMS, ED, and IC settings without disrupting existing processes.

Fourth, contributes to the production of a controlled dataset using clinical procedures of simulated AVPU scoring, in several environments, supporting training and validation of algorithms.

Fifth, it provides scenario-specific implementation models reflecting the operational realities of each environment. These models illustrate how the system adapts to variations in lighting, space, staffing, workflow structure, connectivity, and clinical priorities.

Sixth, it applies Rogers’s Innovation Adoption Curve and the Bass Diffusion Model to forecast the likely adoption trajectory of this technology across the Portuguese NHS. These models provide insight into expected adoption dynamics, highlight critical phases such as the chasm, and underline the importance of robust pilot evidence and integration support. The Bass Diffusion Model parameters applied (innovation coefficient *p* = 0.01, imitation coefficient q = 0.35, market potential M = 111 Portuguese hospitals) require explicit justification. The value *p* = 0.01 reflects the low external-influence adoption rate typical of novel medical-device software; comparable values (0.003–0.015) have been documented for health information technology deployments including electronic prescribing and PACS, as reviewed by Greenhalgh et al.^38^ and Fichman^39^. The value q = 0.35 captures word-of-mouth diffusion within a concentrated professional community; values of 0.3–0.5 are consistent with clinical technology adoption studies where early-adopter champions accelerate peer uptake^40^. M was bounded to the 111 SNS hospitals with declared intermediate care or HDU capacity (2023 data). These choices are informed working assumptions, not empirically calibrated estimates; sensitivity analyses across plausible parameter ranges are provided to bound the projections.

Implications for Clinical Practice: Automated AVPU classification has the potential to improve clinical practice in several ways:

- Enabling more accurate and complete triage documentation/registry;
- Reducing variation in assessment quality between clinicians;
- Supporting early detection of deterioration in wards;
- Improving communication between EMS and hospital teams;
- Enriching NEWS calculations, leading to more reliable escalation decisions.

These improvements may translate into earlier interventions, better patient outcomes, and more efficient use of healthcare resources.

Implementation Considerations. Although technologically feasible, implementation requires addressing several challenges. These include ensuring privacy-preserving processing of video data, integrating the system with existing EHR platforms, training clinical staff, and calibrating hardware to diverse clinical environments. Governance frameworks must clearly define acceptable use, consent requirements, and responsibilities for validation and documentation.

The system must also account for the heterogeneity of hospital infrastructures. Institutions differ in IT maturity, staffing, and readiness for digital transformation, which may affect deployment timelines. Pilot studies should therefore be conducted in representative settings and used to refine the system before broader rollout.

Several limitations of this work should be acknowledged. The development of the model relies on controlled video datasets rather than real-world recordings, due to ethical and logistical constraints. As a result, model performance in clinical environments requires further evaluation. The architectural and adoption analyses, while grounded in available literature and realistic assumptions, remain theoretical until validated through operational pilots. The system’s generalisability to other countries or non-NHS environments may require adaptation. The simulation-to-real transfer gap is the most critical unresolved concern: the dataset consists entirely of healthcare professionals performing AVPU interactions under controlled lighting conditions, and therefore lacks the clinical heterogeneity of genuine emergency patients, including individuals with stroke, acute delirium, pharmacological sedation, severe agitation, or significant facial trauma -- conditions that may substantially alter the facial and motor cues on which the model relies. Prospective quantitative validation -- encompassing per-class accuracy, sensitivity, specificity, macro-averaged F1 score, confusion matrices, and inter-rater agreement (Cohen’s kappa) between model output and expert clinical annotation -- has not been conducted and constitutes the most important prerequisite before any clinical deployment. Future validation studies should also evaluate subgroup performance across skin tones, age groups, and patient positions to detect and mitigate potential algorithmic bias.

Additionally, the use of a single consciousness scale (AVPU) simplifies the design but does not address more granular assessments such as the Glasgow Coma Scale. Future work may explore automated scoring of additional parameters or multimodal integration with other physiological signals. The adoption projections are explicitly scoped to the Portuguese NHS context. Translating them to other healthcare systems requires adaptation of the market potential M, the p coefficient (which varies with national health-IT maturity and regulatory environment), and the q coefficient (which reflects the density of clinical professional networks). Healthcare systems with centralised national digital health programmes may exhibit faster uptake, while fragmented systems with heterogeneous EHR landscapes may see slower diffusion. The framework nonetheless remains transferable in structure, requiring country-specific re-parameterisation as local pilot data become available.

### Future research directions

Several promising avenues of research emerge from this work: (1) Development of larger and more diverse datasets for training and evaluation, including ethically approved clinical recordings; (2) Exploration of multimodal systems combining video, audio, and sensor data to enhance detection accuracy; (3) Extension of automated assessment to NEWS2 Confusion indicator, components of GCS, detection of delirium or sedation states; (4) Implementation of prospective clinical trials to assess real-world impact on triage accuracy, workflow efficiency, and patient outcomes; (5) Economic evaluation of deployment costs and benefits, supporting institutional decision-making; (6) Adaptation of the system for remote triage, telemedicine, or home-monitoring contexts; and (7) Integrate the AVPU automated engine into AAL technologies, bridging partnerships for business scale.

These directions may significantly expand the potential of automated consciousness assessment in modern healthcare systems.

This work provides a comprehensive foundation for the development, deployment, and diffusion of automated AVPU classification across emergency and hospital care. By addressing both technological and operational challenges, the work contributes to the broader goal of advancing digital transformation in healthcare. Automated consciousness assessment represents an important step toward more objective, continuous, and interoperable monitoring, supporting clinicians in delivering timely and effective care.

The proposed architecture and adoption analysis provide a roadmap for future implementation and evaluation. With appropriate investment in research, clinical validation, and integration support – Consc.ia - the automated AVPU system may become a standard component of emergency care and patient safety frameworks.

## Supplementary Information

Below is the link to the electronic supplementary material.

Supplementary Material 1

## Data Availability

The annotated video dataset generated and analysed during the current study (used to train the AI models) is not publicly available due to ethical and privacy restrictions. The data are available from the corresponding researcher upon reasonable request and subject to additional ethics approval. Requests should be directed to Pedro Faria (pedro.moises.faria@gmail.com).
